# Integrative Approaches in Computational Biomedical Imaging 2013

**DOI:** 10.1155/2014/757380

**Published:** 2014-01-27

**Authors:** Huafeng Liu, Pengcheng Shi, Yunmei Chen

**Affiliations:** ^1^State Key Laboratory of Modern Optical Instrumentation, Zhejiang University, Hangzhou 310027, China; ^2^B. Thomas Golisano College of Computing and Information Sciences, Rochester Institute of Technology, Rochester, NY 14623, USA; ^3^Department of Mathematics, University of Florida, 458 Little Hall, Gainesville, FL 32611-8105, USA

Biomedical imaging has continued to play increasingly important roles in clinical practice and research activities. While the enormous amount of imaging data provides observations on the organisms of interest, these data have to be properly analyzed in order to reveal the *insights* into the biological and physiological processes.

This special issue, following the very successful one in 2012, provides a snapshot of the latest and emerging computational biomedical imaging technologies and their applications in clinics and research. From the 20 submissions that have gone through the peer-review process, the guest editors have selected six papers covering some very interesting and timely topics, from forensic medicine to cardiovascular risk assessment.

The work of W. Lin et al. attempts to predict cardiovascular risk by using a combination of physiological parameters, including blood pressure, electrocardiogram, arterial stiffness, ankle-brachial blood pressure index, and blood glucose carrying valuable information. This paper provides the current status on the medical devices for physiological measurements and presents various points of view on potential implications for promoting cardiovascular disease prevention and treatment in the future.

Ultrasound imaging is the focus of the next two papers. The paper by J. Xia et al. presents an original concept for the visualization of temperature distribution in tissue building upon changes in ultrasonic backscattered energy. This approach visualizes temperature map in tissues during nonuniform heating without the need of tracking and compensation of the echo shift. W. Cong et al. describe an intriguing platform to simulate ultrasound based on CT images, which has the potential to facilitate ultrasound guided navigation in clinical practice.

The paper by R. Xiao et al. focuses on vessel tracking for coronary artery identification. The system integrates multiscale Hessian information and discriminates the connecting relationship of the tracked ridge points in order to automatically track vessel from X-ray angiograms. This work has demonstrated its capability through clinical evaluation.

H. Jiang et al. present clinically important work on recognizing liver cancer from abdominal CT images by using multi-instance learning and support vector machine, which aims to improve the efficiency for liver cancer detection. The other paper of the special issue explores a novel application in forensic medicine and the determination of sex from 3D digital skulls using statistical shape model. The method has been tested on 208 skulls with very promising results.

We appreciate the many high quality submissions to this special issue from leading researchers in computational biomedical imaging.

*Huafeng Liu* *Huafeng Liu* *Pengcheng Shi* *Pengcheng Shi* *Yunmei Chen* *Yunmei Chen*

